# Free-Standing and Heteroatoms-Doped Carbon Nanofiber Networks as a Binder-Free Flexible Electrode for High-Performance Supercapacitors

**DOI:** 10.3390/nano9091189

**Published:** 2019-08-22

**Authors:** Xiaona Yan, Hanjing You, Wei Liu, Xiaodong Wang, Dezhen Wu

**Affiliations:** State Key Laboratory of Chemical Resource Engineering, Beijing University of Chemical Technology, Beijing 100029, China

**Keywords:** supercapacitor, flexible electrode, carbon nanofiber, electrospinning, polyimide

## Abstract

Flexible and heteroatoms-doped (N, O and P) activated carbon nanofiber networks (ACFNs) have been successfully prepared with a mixture of polyamic acid (PAA) and poly(diaryloxyphosphazene) (PDPP) as a solution through electrospinning, followed by a heat post-treatment. The resultant heteroatoms-doped ACFNs can be used as binder-free electrodes for high-performance flexible supercapacitors (SCs) due to lightweight, three-dimensional open-pore structure and good mechanical strength. Despite its surface area being lower than 130.6 m^2^·g^−1^, the heteroatoms-doped ACFNs exhibited a high heteroatoms (N, O and P) content of 17.9%, resulting in a highly specific capacitance of 182 F·g^−1^ at a current density of 1 A·g^−1^ in 6 M KOH electrolyte in a two-electrode cell and an excellent rate capability of 74.7% of its initial capacitance from 1 A·g^−1^ to 10 A·g^−1^ under the mass loading of 1.5 mg·cm^−2^. The electrical double-layer (EDL) capacitance and pseudocapacitance can be easily decoupled in the heteroatoms-doped mesoporous ACFNs. SCs device based on heteroatoms-doped ACFNs exhibited a high energy density of 6.3 W·h·kg^−1^ with a power density of 250 W·kg^−1^, as well as excellent cycling stability with 88% capacitance retention after 10,000 charge–discharge cycles. The excellent electrochemical performance was attributed to the mesoporous structure of ACFNs and pseudocapacitive heteroatoms.

## 1. Introduction

The development of portable electronics such as roll-up displays, hand-held devices and wearable personal multi-media has raised urgent needs for flexible and lightweight energy storage devices [1,2,3,4,5,6,7,8,9]. Supercapacitors (SCs), which have superior power density, fast charge–discharge rates, long cycle life and safety, have demonstrated their potential applications in hybrid vehicles, backup power, personal multimedia, and flexible electronics [10,11,12]. Conventional SCs are composed of current collectors, an outer case and positive and negative electrodes in an electrolyte separated by an ion transport layer [13]. Moreover, the employment of inactive components including conductive additives, binders and current collectors makes the conventional SCs so thick, heavy and rigid in order to meet the practical requirements for portable devices [14]. Therefore, there is an urgent need for developing flexible SCs. A flexible electrode is a key component for flexible SCs. Flexible electrodes can be divided into three types according to the review of Liu et al. [13], which include fiber-like, paper-like and three-dimensional porous flexible electrode. Among paper-like flexible electrodes, free-standing activated carbon nanofiber networks (ACFNs) produced by electrospinning have been outstanding in flexible SCs due to a large specific surface area, excellent mechanical flexibility and good electrical conductivity [14]. Besides, it is apparent that free-standing ACFNs do not need additional substrates and current collectors, greatly reducing the weight of electrodes and saving the trouble of a coating process.

Generally, SCs are classified into two categories: electrochemical double-layer (EDL) capacitors and pseudocapacitors according to different energy storage mechanisms [15,16,17]. EDL capacitors store energy via electrostatic attraction between charges accumulated at the electrode–electrolyte interface. Pseudocapacitors store energy by fast and reversible faradic processes on/near electrode surface. Compared with pseudocapacitors, EDL capacitors possess better rate capability and longer durability and are the most common SCs in practice. Generally, pure carbon electrode materials for EDL capacitors including carbon onion, carbon nanotube, graphene and carbon nanofiber hardly acquire a specific capacitance more than 150 F·g^−1^ so far. The two energy storage mechanisms for SCs can function simultaneously depending on the nature of the electrode materials, so an effective combination of EDL capacitance and pseudocapacitance could enhance the overall capacitance of SCs.

Pseudocapacitive materials normally including metal oxides and electrical conductive polymers have been extensively used to deliver highly specific capacitance. Nevertheless, the commercial utilization of pseudocapacitive materials is confined by the low conductivity and high price [18,19]. Recently, heteroatoms-doped carbon materials through the introduction of foreign elements have attracted growing attention due to their potential applications in energy storage. Single or dual doping chemistry of heteroatoms, such as nitrogen (N), sulfur (S), phosphorus (P) and boron (B), is promising to improve the capacitive performance due to the additional pseudocapacitance caused by the formation of electrochemical active sites [20,21,22]. Nitrogen-doped electrode materials possess attractive pseudocapacitance from faradic redox reactions between the nitrogen functional groups and the ions in electrolytes. Moreover, the nitrogen in the carbon framework can improve the electrical conductivity and wettability of the interface between the electrolyte and the electrode [18,19,23,24]. The doping of phosphorous can induce pseudocapacitance [25] and also enhance wettability and electrical double-layer capacitance [26]. Polyimide (PI) as a carbon precursor has been widely used to fabricate nitrogen and oxygen-enriched carbon nanofiber networks for flexible electrode materials due to its relatively high carbon yield and high nitrogen content [27]. Recently, Liu et al. [28] explored poly(diaryloxyphosphazene) (PDPP) as a carbon precursor enriched with nitrogen, phosphorous and oxygen heteroatoms and also proposed a type of in-situ heteroatoms-doping method for preparing porous carbon materials. PI and PDPP are both excellent carbon precursors with enriched nitrogen, phosphorous and oxygen heteroatoms and can be combined simply through an electrospinning technique.

Electrospinning can produce free-standing nanofiber nonwoven networks as a promising and straightforward technique, and the resulting nanofiber networks can be used as both electrodes and separators for flexible energy storage devices [29,30,31]. In this study, electrospining technique was employed to prepare the networks based on PI/PDPP nanofiber firstly and then the carbonization and activation processes were adopted to fabricate free-standing and flexible ACFNs enriched with N, O, P heteroatoms. Electrospinning not only provides a simple preparing method for flexible electrodes but also offers the combination of different carbon precursors with different doping elements, thus incorporating EDL capacitance and pseudocapacitance effectively. Controlling the nitrogen and phosphurous content was realized by varying mass fraction of PDPP in the spinning solution. With increasing the mass content of PDPP, the specific capacitance of flexible SCs prepared from ACFNs doped with N, O, P heteroatoms obtained an apparent increase due to pseudocapacitance and increased mesoporous structure.

## 2. Materials and Methods

### 2.1. Synthesis of Heteroatoms-Doped Activated Carbon Nanofiber Networks (ACFNs)

Thepolyamic acid (PAA) solution was firstly synthesized from biphenyl tetracarboxylic dianhydride (BPDA) and p-Phenylenediamine (PPD) in *N,N*-dimethyll formamide (DMF). The monomer BPDA and PPD were purchased from Beijing Sinmaya Chemicals Co., Ltd. and purified by sublimation before use. The solvent DMF was purchased from DuPont Co. and used after distillation. Poly(diaryloxyphosphazene) (PDPP) was prepared in our lab, and the process of preparation was introduced in our previous reports [28]. Varied PAA/PDPP mixed solutions in DMF were then obtained by adjusting the mass fraction of PDPP, and the mass fraction of PDPP was 3.9 wt %, 6.4 wt %, 10.3 wt % and 12.9 wt % respectively. Subsequently, PAA/PDPP mixed solution was electrospun to prepare PAA/PDPP nanofiber nonwoven networks, and corresponding polyimide/PDPP (PI/PDPP) nanofiber networks were prepared through thermal treatment at 300 °C for 2 h. PI/PDPP nanofiber networks sandwiched between graphite sheets were then carbonized at 800 °C for 2 h with a heating rate of 5 °C/min in tube furnace with nitrogen protection to prepare carbonized nanofiber networks. KOH was used as an activating agent. Varied carbonized nanofiber networks were rinsed in 4 M KOH solution for 0.5 h and then dried in a vacuum oven. The resultant dry carbonized nanofiber networks were activated ultimately at 600 °C for 2 h with a heating rate of 5 °C/min in tube furnace with nitrogen protection to prepare ACFNs. The ACFNs were neutralized by hydrogen nitrate and washed by a large content of water and then dried in vacuum oven. The corresponding ACFNs with different contents of PDPP were referred to as ACF-3.9% PDPP, ACF-6.4% PDPP, ACF-10.3% PDPP and ACF-12.9% PDPP, respectively.

### 2.2. Characterization

The morphology of the samples was characterized by a field emission scanning electron microscope (FE-SEM, HITACHI, S-7700, Tokyo, Japan). The structure was characterized on a wide-angle X-ray diffractometer (WAXD, D8 FOCUS, Bruker, Karlsruhe, Germany, Cu Ka, λ = 0.154 nm). Raman spectra were obtained with a Renishaw RM1000 confocal microscope. The spectra were collected in a region of 500–4000 cm^−1^ with an excitation wavelength of 514 nm. The surface elements composition and chemical status of the samples were analyzed by X-ray photoelectron spectroscopy (XPS, K-Alpha, Thermo Fisher Scientific, Waltham, MA, USA). The specific Brunauer-Emmett-Teller (BET) surface area and pore-size distribution of the samples were measured on a static volumetric instrument (Micromeritics, ASAP 2460, Norcross, GA, USA) by nitrogen adsorption at 77 K.

Electrochemical measurements were performed on a two-electrode system. The ACFNs were used as working electrodes directly without any binder and conductive agent. The filter paper, which served as a separator, was sandwiched between two working electrodes with almost the same mass. The density per unit area of each working electrode is about 1.5 mg·cm^−2^.) KOH aqueous solution (6.0 mol/L) was used as the electrolyte in electrochemical tests. The cyclic voltammetry (CV), galvanostatic charge/discharge (GCD) and electrochemical impedance measurements were carried out on CHI 660D electrochemical workstation. The electrochemical impedance measurement was performed in a frequency range from 100 kHz to 0.01 Hz with an AC amplitude of 10 mV.

## 3. Results and Discussion

### 3.1. Morphology and Structure

**Figure 1:**
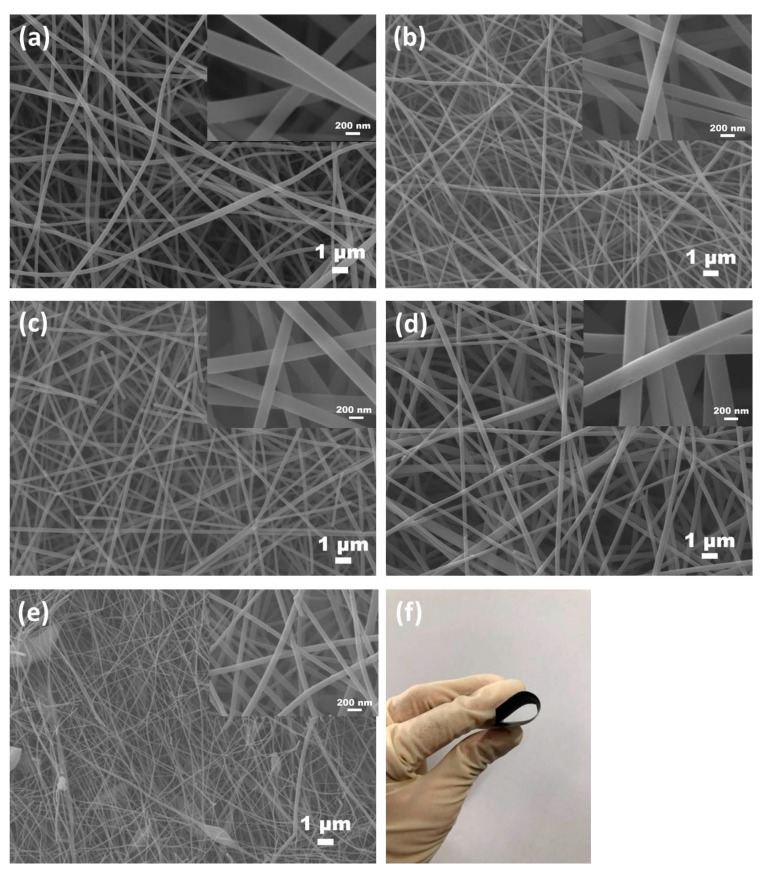
SEM images of (**a**) ACF-0; (**b**) ACF-3.9% PDPP; (**c**) ACF-6.4% PDPP; (**d**) ACF-10.3% PDPP; (**e**) ACF-12.9% PDPP; and (**f**) optical image of ACF-10.3% PDPP.

**Figure 2:**
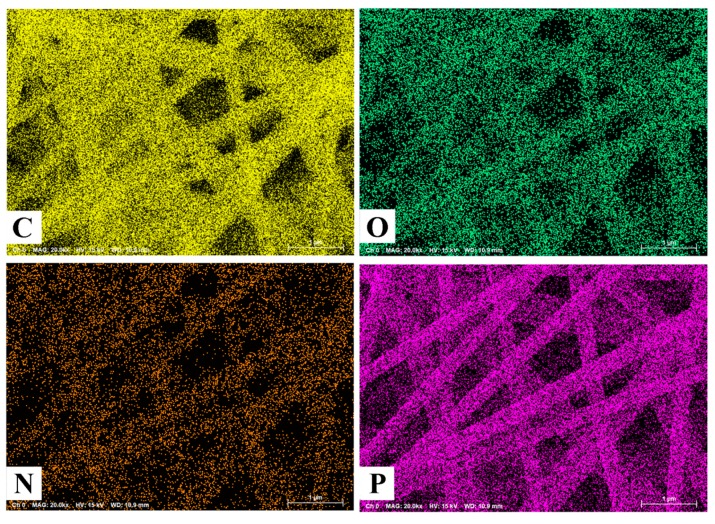
SEM mapping results of ACF-10.3% PDPP.

Figure 1 shows the typical fibrous morphology of heteroatoms-doped ACFNs with different contents of PDPP. Activated carbon nanofibers (ACFs) in all samples are long, consecutive and construct a type of three-dimensional fibrous network, which is favorable to increase the conductivity and flexibility. The diameter of ACFs decreases with increasing the content of PDPP due to the lower carbon yield of PDPP. The diameters of ACFs with different contents of PDPP are uniform except the sample of ACF-12.9% PDPP. A few fibers of ACF-12.9% PDPP sample have beads due to the strengthened hydrogen bond interaction with increasing the content of PDPP. When the content of PDPP exceeds 12.9 wt%, more beads are introduced into the fibers and electrospinning is hard to be proceeded, so 12.9 wt% is taken as the highest content of PDPP. All ACFNs display outstanding flexibility, which could withstand large bending, as shown in Figure 1f. PAA and PDPP molecules are distributed uniformly in fibers after the electrospinning process so the heteroatoms are well-distributed accordingly, which can be seen from SEM mapping in Figure 2. Uniform heteroatoms doping could improve electrochemical performance of the carbon materials.

**Figure 3:**
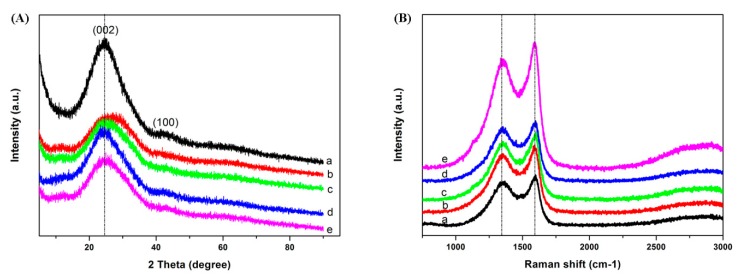
(**A**) XRD patterns and (**B**) Raman spectra of (**a**) ACF-0; (**b**) ACF-3.9% PDPP; (**c**) ACF-6.4% PDPP; (**d**) ACF-10.3% PDPP; and (**e**) ACF-12.9% PDPP.

The XRD patterns of the ACFNs with different contents of PDPP are shown in Figure 3a, which shows similar diffraction features. There are two wide diffraction peaks near 25° and 43°, assigned to the graphitic (002) and (100) planes, respectively, which means all the ACFNs are mainly amorphous carbon [32]. After the addition of PDPP, it is evident that the intensity of the (002) diffraction peak decreases and its full width at half maximum turns broader, demonstrating that the crystalline structure is destructed by mixing of the PAA and PDPP macromolecules. To further analyze the degree of graphitization of all the ACFNs with different contents of PDPP, Raman spectroscopy was applied. As Raman spectra of all ACFNs with different contents of PDPP shown in Figure 3b, there are two peaks around 1352 cm^−1^ (D-band) and 1590 cm^−1^ (G-band). The D-band stands for disorder and defects, while the G-band represents the in-plane vibration of the sp^2^ carbon atom [33,34,35]. The ratio of the relative intensity of the D-band and G-band (I_D_/I_G_) is proportional to the content of defect sites in carbon materials. The value of I_D_/I_G_ for ACFNs with different contents of PDPP is shown in Table 1 and increases from 0.88 (ACF-0) to 0.94 (ACF-10.3% PDPP) then decreases to 0.91 (ACF-12.9% PDPP). After the addition of PDPP, the value of I_D_/I_G_ decreases overall, suggesting a less ordered crystallite structure, which is in good agreement with the wide-angle XRD profiles.

**Figure 4:**
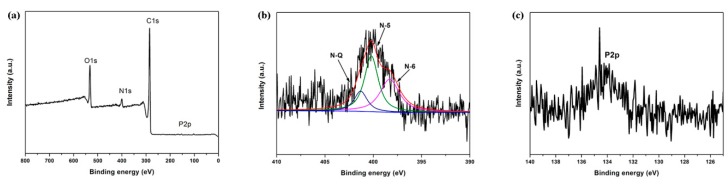
(**a**) XPS pattern; (**b**) high-resolution spectra for N1s and (**c**) spectra of P2p of ACF-10.3% PDPP.

XPS analysis was conducted to investigate the contents and states of heteroatoms of ACFNs with different contents of PDPP. The survey XPS spectra and high-resolution spectra of N1s and P2p are shown in Figure 4, and the summary elemental analysis for ACFs-10.3% PDPP sample is presented in Table 2. Survey and high-resolution spectra have shown a certain content of heteroatoms of N, P and O elements contained in the ACFNs. The identification of heteroatoms is advantageous as heteroatoms can considerably contribute to an additional pseudocapacitance as well as improved wettability. After deconvolution of the peak for N1s, different atomic environments were found to exist. Based on the elemental analysis from the survey scan and the individual atomic environments from the narrow scan deconvolution, atomic percent were calculated for specific functionalities.

The high-resolution spectra of heteroatoms give valuable insight into the functional groups present on the surface of heteroatoms-doped ACFNs, and different functionalities are identified based on their binding energies. The N1s spectra indicate that the residual nitrogen atoms in the ACFs-10.3% PDPP sample are in three different environments: pyridinic (N-6, 398.3 eV), pyrrolic/pyridine (N-5, 400.2 eV) and quaternary (N-Q, 401.3 eV) [36,37,38]. N-6 and N-5, which are supposed to locate at the edges of graphene layers, contributed to pseudocapacitance according to previous studies [39,40]. Moreover, the content of N-6 and N-5 (3.5 at %) accounts for 84.5% in the nitrogen content (4.14 at %) calculated from XPS analysis and is predominant in the product. The content of the P element is 0.45% (shown in Table 2), which is much lower than the content of N element, indicating the weak pseudocapacitance contributed to from the P element. In a word, N, P and O-doped flexible ACFNs could be prepared in situ by direct carbonization and activation of electrospun PAA/PDPP nanofiber networks. The heteroatoms doped into carbon materials can not only contribute pseudocapacitance but also improve electrical conductivity and wettability, which is beneficial to obtain excellent capacitive performance [41,42].

To trace the content change of N, P and O heteroatoms in nanofiber networks through different thermal treatments, the relative element contents of nanofiber networks after carbonization and activation are listed in Table 3. After the carbonization process, N, P and O elemental content decreases dramatically due to high-temperature pyrolysis, and some acid materials related to phosphoric acid are produced. After KOH activation, the elemental content of N and P decreases further but more slowly due to the neutralization of acid materials and KOH; then microporous and mesoporous structures are introduced accordingly. The content of surface oxygen increases, which usually comes from the residual stable oxygen of the precursor which endured high temperature pyrolysis and introduced oxygen during activation by the activation agent KOH.

Elemental content analysis of ACFNs with different contents of PDPP is shown in Table 4. With increasing the content of PDPP, the relative elemental content of P in heteroatoms-doped ACFNs increases first and then reaches a steady value. The more PDPP that is doped, the more acid materials are produced. After KOH activation, acid materials are all neutralized by KOH, and the relative elemental content of P almost reaches a steady value. The relative elemental content of N exhibits a decreasing tendency probably due to the catalysis degradation effect on N element to NH_3_ and NOx from P element.

**Figure 5:**
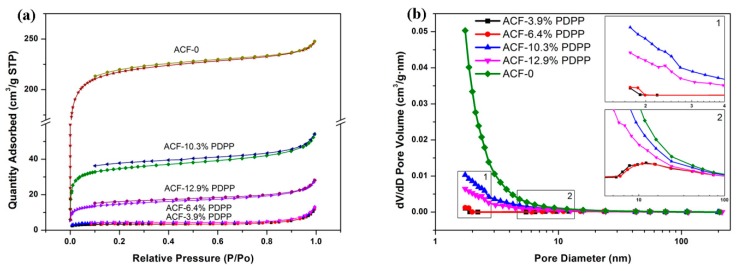
(**a**) Nitrogen adsorption and desorption isotherms, and (**b**) pore size distribution of ACF-0, ACF-3.9% PDPP, ACF-6.4% PDPP, ACF-10.3% PDPP, and ACF-12.9% PDPP samples.

**Figure 6:**
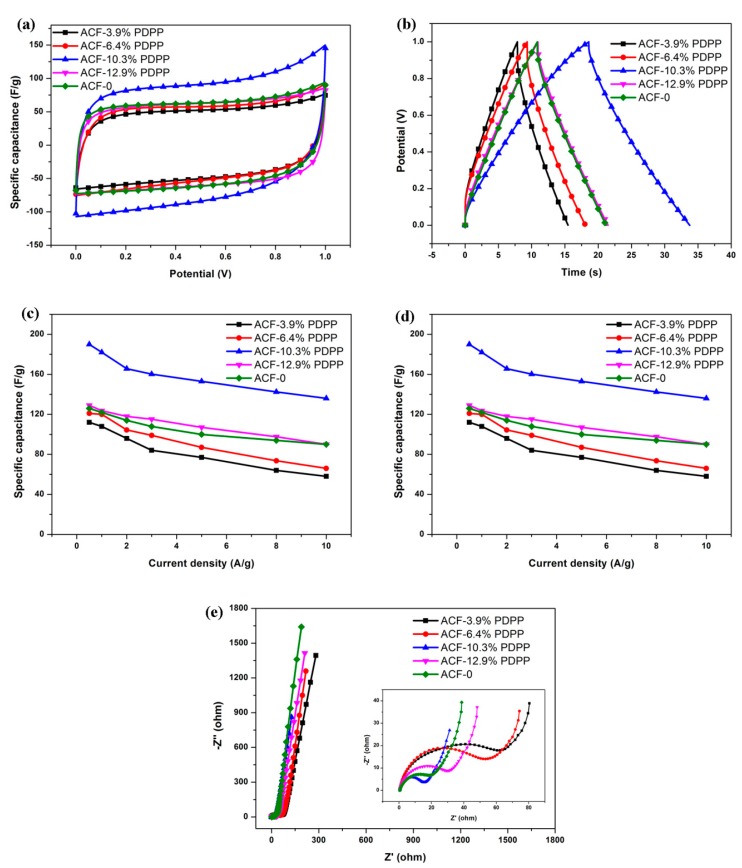
(**a**) CV curves of all samples at a scan rate of 30 mV·s^−1^; (**b**) GCD curves of all samples at current densities of 5 A·g^−1^; (**c**) specific capacitance of each sample at different current densities (0.5–10 A·g^−1^); (**d**) Ragone plots as a function of current densities (0.5–10 A·g^−1^) of symmetric supercapacitors; and (**e**) Nyquist plots of all samples in the frequency range of 100 kHz and 0.01 Hz. The inset in (e) is the magnified region.

The nitrogen adsorption/desorption isotherms, pore size distribution plots and the textural properties of the ACFNs with different contents of PDPP are shown in Figure 5 and Table 5, respectively. The sample of ACF-0 shows a high nitrogen uptake at relative pressure (P/P_0_) below 0.1 and presents the type-I sorption isotherm according to the classification of IUPAC [43], indicating the microporous characteristic, and the average diameter of micropores is 1.8 nm. Moreover, there is no hysteresis loop in the sorption isotherm of ACF-0, indicating no mesopores formed, which can also be identified from Figure 6b. For the samples of ACFNs with different contents of PDPP, the sorption isotherms all showed typical characteristics of type-IV. Moreover, there is a hysteresis loop which appeared in the medium pressure region, and the hysteresis loop is assigned to be the H4 type, indicating the formation of mesopores. Based on isotherms analysis above, the addition of PDPP is beneficial to form a mesoporous structure, which is favorable for transportation of electrolyte ions in carbon materials. When a small content of PDPP (ACF-3.9% PDPP) is added, the BET surface area decreases dramatically from 814.2 m^2^·g^−1^ to 12.6 m^2^·g^−1^ and the average pore diameter increases from 1.8 nm to 3.3 nm, suggesting the disappearance of a large content of micropores and appearance of a few mesopores. When the content of PDPP increases, the BET surface area increases and reaches the maximum value of 130.6 m^2^·g^−1^ when the content of PDPP is 10.3 wt %, and then decreases. PDPP molecules are easy to shrink and accumulate to form immense carbon bulk during the carbonization process and only a small number of micropores form in the heteroatoms-doped ACFNs, which explains the dramatic decrease of the BET surface area. With increasing the content of PDPP, part of the micropores enlarges to mesopores due to the emission of pyrolysis gases caused by the decomposition of the polymer. Although the BET surface area decreases, the specific capacitance increases mainly due to the reasonable hierarchical pore structure (micropores and mesopores). Mesopores can accelerate the transportation of electrolyte ions, and the electrochemical property of this hierarchical porous structure can be improved accordingly [44,45,46].

### 3.2. Electrochemical Performance

To evaluate the electrochemical performance of all ACFNs as supercapacitor electrodes, the cyclic voltammetry (CV), galvanostatic charge/discharge (GCD) and electrochemical impedance spectroscopy (EIS) tests were performed. The electrochemical performance of all ACFNs in 6 M KOH aqueous electrolyte was analyzed in a two-electrode system.

Figure 6a shows CV curves of all samples at a scan rate of 30 mV·s^−1^ and the voltage window is 0–1 V. It can be observed that ACF-0 appears to have a quasi-rectangular shape, indicating the typical characteristic of an EDL capacitor. Heteroatoms-doped ACFNs with different contents of PDPP present slightly deformed rectangular-shaped CV curves, suggesting the presence of heteroatom functional groups. Among these heteroatoms-doped ACFNs samples, ACF-10.3% PDPP shows the largest area covered by CV curve, which means ACF-10.3% PDPP has the largest specific capacitance. With increasing the content of PDPP, the specific capacitance increases to the highest value then tends to decrease. It is implied that there exists an optical heteroatom content to obtain the best electrochemical properties.

Figure 6b shows the GCD curves of all samples at a current density of 5 A·g^−1^. The shapes of all the GCD curves are closely linear and symmetrical, indicating a typical characteristic of an EDL capacitor. It is evident that ACF-10.3% PDPP has achieved the maximum specific capacitance according to discharge plots. The specific capacitance is up to 153 F·g^−1^ at 5 A·g^−1^ and has improved efficiently compared with 77 F·g^−1^ of ACF-0 at 5 A·g^−1^. The relative highly specific capacitance can be mainly ascribed to the hierarchical porous structure and the disordered microscopic distribution of heteroatoms in activated carbon nanofibers, which can be well explained by the results from the nitrogen adsorption–desorption and Raman measurements. The nitrogen adsorption–desorption measurements have shown that ACF-10.3% PDPP has the largest BET surface area and mesoporous structure. Besides, the I_D_/I_G_ value of ACF-10.3% PDPP in Raman tests is the largest too, indicating the most disordered distribution of heteroatoms and graphitic structure.

Figure 6c shows the specific capacitance at different current densities for all samples. The specific capacitance is calculated based on discharge time in the GCD curve. For all samples, the specific capacitance decreases with the increasing current density. The specific capacitance of ACF-10.3% PDPP at each current density is the highest among all the samples, which exhibits the optimized doping content of PDPP is 10.3 wt %. The highest capacitance of ACF-10.3% PDPP is 182 F·g^−1^, which is achieved at a current density of 1 A·g^−1^. ACF-10.3% PDPP also presents outstanding rate capability. Its specific capacitance still reaches 136 F·g^−1^ at the current density of 10 A·g^−1^, which is about 75% of the specific capacitance at 1A·g^−1^.

Figure 6d exhibits the energy density (E, W·h·kg^−1^) as a function of power density (P, W·kg^−1^) for all samples. Energy density and power density of the supercapacitor can be calculated from the following equations [28,47,48]:

Cs = 2(I ∆t)/(m ∆V)(1)

E = 0.5 Cs (∆V)^2^ (2)

P = 3600E/∆t(3)

where, Cs, I, ∆t, m, and ∆V represent the specific capacitance (F·g^−1^) of the supercapacitor, the discharge current (A), the discharge time (s), the mass of activated materials for a single electrode, and the voltage (V) after an IR drop, respectively. ACF-10.3% PDPP is shown to have the highest energy density at each power density. The energy density of ACF-10.3% PDPP supercapacitor is 6.3 W·h·kg^−1^ at a power density of 250 W·kg^−1^ (1 A·g^−1^).

Figure 6e shows the electrochemical impedance spectroscopy of all samples in a frequency range of 100 kHz to 0.01 Hz, and their Nyquist plot shapes are similar. In the low frequency region, all of the samples doped with PDPP show a nearly vertical curve, suggesting a fast ion diffusion and ideal capacitive behavior [27,49]. At high frequency, the intersection at the real part represents the internal resistance, which is almost the same for all samples. The semicircle region in the Nyquist plot accounts for the charge transfer resistance (R_CT_). The R_CT_ of ACF-10.3% PDPP electrode is the smallest, reflecting the most channels and surfaces for charge transportation.

**Figure 7:**
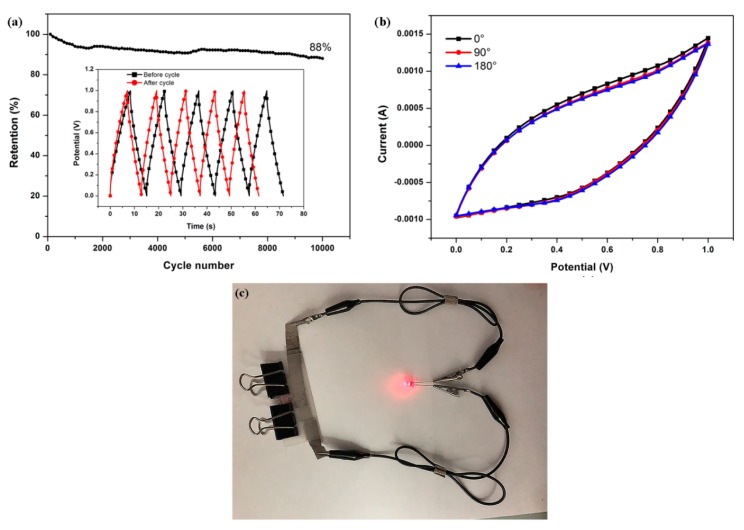
Electrochemical performance of ACF-10.3% PDPP-based symmetric supercapacitor: (**a**) long-term cycle stability test at a current density of 10 A·g^−1^; (**b**) CV curves of ACF-10.3% PDPP bended by different angles (0°, 90° and 180°) at a scan rate of 100 mV·s^−1^; and (**c**) digital photo images of a red LED powered by three devices in series.

The ACF-10.3% PDPP electrode presents an excellent cycling stability especially when pseudocapacitance exists, and the capacitance remains at 88% after 10,000 cycles of galvanostatic charge/discharge at 10 A·g^−1^ (Figure 7a) in a two-electrode cell. The good cycling stability of ACF-10.3% PDPP is mainly ascribed to the hierarchical porous structure and in-situ doped N, O, P heteroatoms dispersed evenly in the carbon bone. Good cycling stability is a crucial parameter for electrode materials in practical applications. Besides, the electrode prepared from ACF-10.3% PDPP exhibits excellent flexibility, which is shown in Figure 7b. CV curves at 100 mV·s^−1^ under different bending angles were tested to evaluate the flexibility of the device. The CV curves have shown almost the same shape with negligible deformation under different bending conditions. The area covered by the CV curves under different bending angles is almost unchanged, suggesting its significant flexibility and mechanical stability. In practical applications, the working voltage and energy of the single device is limited; many supercapacitors are required to be connected in serials to extend operating voltage and energy storage. The assembly of three devices based on ACF-10.3% PDPP in series could power a red light-emitting-diode (LED) well (Figure 7c), confirming its great potential for practical applications in flexible energy storage.

## 4. Conclusions

A flexible, free-standing and heteroatoms-doped ACFNs was prepared with a mixture of PAA and PDPP as a solution through electrospinning, followed by heat post-treatment. The heteroatoms-doped ACFNs obtained unchanged capacitance performance under different bending angles (0°, 90° and 180°), confirming excellent mechanical flexibility. The remarkable flexibility and free-standing characteristics make the heteroatoms-doped ACFNs functionalize as an electrode alone, without the need to add a conductive agent and binder, which can reduce contact resistance. When the content of PDPP was 10.3 wt %, the maximum specific capacitance was achieved. The specific capacitance of ACF-10.3% PDPP is 182 F·g^−1^ at a current density of 1 A·g^−1^ in 6 M KOH aqueous electrolyte, and the excellent specific capacitance is contributed to by the combination of EDL capacitance and pseudocapacitance. The mesoporous structure caused by the addition of PDPP improved EDL capacitance, and heteroatoms (N, O, P) contributed pseudocapacitance. The ACF-10.3% PDPP also shows good cycling stability, and the capacitance retention is 88% after 10,000 cycles of charge/discharge process at the current density of 10 A·g^−1^.The ACF-10.3% PDPP-based supercapacitor can deliver a high energy density of 6.3 W·h·kg^−1^ when the power density is 250 W·kg^−1^. Besides, a red light-emitting-diode (LED) can be powered well by three supercapacitors in series, indicating a promising application in flexible energy storage. All these excellent electrochemical performances makes heteroatoms-doped mesoporous ACFNs a promising candidate for flexible supercapacitor electrode materials.

## Figures and Tables

**Table 1:** I_D_/I_G_ data from Raman spectra of (**a**) ACF-0, (**b**) ACF-3.9% PDPP, (**c**) ACF-6.4% PDPP, (**d**) ACF-10.3% PDPP; and (**e**) ACF-12.9% PDPP.

Sample	(a) ACF-0	(b) ACF-3.9% PDPP	(c) ACF-6.4% PDPP	(d) ACF-10.3% PDPP	(e) ACF-12.9% PDPP
I_D_/I_G_	0.88	0.90	0.91	0.94	0.91

**Table 2:** XPS analysis of ACF-10.3% PDPP sample and corresponding relative contents of nitrogen and phosphorus species from core level XPS spectra.

C	O	N	P	N1s (at %)		
(at %)				N-6	N-5	N-Q
82.09	13.32	4.14	0.45	1.58	1.92	0.64

**Table 3:** Element content of fibrous networks prepared from PAA-12.9% PDPP through different thermal treatments.

	Elements	C (at %)	O (at %)	N (at %)	P (at %)
Sample					
PI-12.9% PDPP		77.38	15.25	6.72	0.65
CF-12.9% PDPP		85.9	10.14	3.41	0.55
ACF-12.9% PDPP		81.67	14.92	2.98	0.44

**Table 4:** Element content of ACFNs prepared from PAA-PDPP fibrous networks with different contents of PDPP.

	Elements	C (at %)	O (at %)	N (at %)	P (at %)
Sample					
ACF-0		82	12.34	5.66	
ACF-3.9% PDPP		82.78	11.32	5.65	0.25
ACF-6.4% PDPP		81.32	13.98	4.25	0.45
ACF-10.3% PDPP		82.09	13.32	4.14	0.45
ACF-12.9% PDPP		81.67	14.92	2.98	0.44

**Table 5:** Surface texture properties of ACFNs prepared from PAA/PDPP fibrous networks with different ratios.

Sample	S_BET_ (m^2^·g^−1^)	S_micro_ (m^2^·g^−1^)	S_ext_ (m^2^·g^−1^)	V_total_ (m^2^·g^−1^)	APD ^a^ (nm)
ACF-0	814.2	672.7	141.5	0.37	1.8
ACF-3.9% PDPP	12.6	9.7	2.9	0.011	3.3
ACF-6.4% PDPP	16.5	12.8	3.7	0.012	2.9
ACF-10.3% PDPP	130.6	90.6	40	0.073	2.2
ACF-12.9% PDPP	54.6	30.2	24.4	0.035	2.6

^a^ APD means Average Pore Diameter.
